# Dataset of 16S ribosomal DNA sequences of bacteria isolated from marine red algae *Kappaphycus alvarezii*

**DOI:** 10.1016/j.dib.2021.107784

**Published:** 2022-01-01

**Authors:** Rennielyn Rupert, Kenneth Francis Rodrigues, Harry Lye Hin Chong, Wilson Thau Lym Yong

**Affiliations:** a Biotechnology Research Institute, Universiti Malaysia Sabah, Jalan UMS, Kota Kinabalu, Sabah 88400, Malaysia; b Faculty of Science and Natural Resources, Universiti Malaysia Sabah, Jalan UMS, Kota Kinabalu, Sabah 88400, Malaysia

**Keywords:** 16S rDNA gene sequencing, Bacterial community, Red algae, *Kappaphycus alvarezii*, Persistent bacteria

## Abstract

The data provided in the article contains bacterial community profiles present on the surface of red algae (*Kappaphycus alvarezii*) isolated directly after collection and after 30 days of cultivation in a closed circulation system. The explants of *Kappaphycus alvarezii* were cultivated in a laboratory setting under controlled growth conditions for 30 days in order to determine bacteria that could adapt to controlled culture conditions. Amplification and sequencing of bacterial 16S rDNA amplicon were performed on bacterial isolates associated with the seedlings. The 16S rDNA gene sequences were analyzed, trimmed, and assembled into contigs using DNA Baser Sequence Assembler (V5) software. Taxonomic identification for the assembled sequences was achieved using the online BLAST (blastn) algorithm, and the construction of a phylogenetic tree was performed using the MEGA7 software. The data reveals a distinct set of microbial variations between day one and day 30. The phylogenetic tree depicts four major clusters, *Vibrio, Pseudoalteromonas, Alteromonas*, and *Bacterioplanes* resident on the surface of the *K. alvarezii*. Comparison between these two bacterial groups provides evidence of the persistent marine bacteria that adapt to the long-term culture in closed circulation systems. Raw data files are available at the GenBank, NCBI database under the accession number of MZ570560 to MZ570580.

**Specifications Table**

**Table:** 

Subject	Microbiology
Specific subject area	Microbiome analysis in red algae
Type of data	Table and figure
How data were acquired	Amplification of 16S rDNA and sequencing of bacterial 16S rDNA amplicons of bacterial isolates from red algae, *K. alvarezii*
Data format	Raw and analyzed
Parameters for data collection	Bacterial isolates were isolated directly from the red algae using a culture-dependent method.
Description of data collection	The bacterial swab samples were collected directly from the surface of red algae on day one and day 30 of seaweed cultivation in triplicates. The 16S rDNA gene sequences were trimmed and assembled using the DNA Baser Sequence Assembler (V5) software and identified using the online BLAST (blastn) algorithm. The phylogenetic tree was constructed using the MEGA7 software based on the assembled 16S rDNA sequences.
Data source location	Seedlings of red algae, *K. alvarezii*, were cultivated in the seaweed cultivation laboratory, Biotechnology Research Institute, Universiti Malaysia Sabah, Malaysia. Institution: Universiti Malaysia Sabah City/Town/Region: Sabah Country: Malaysia
Data accessibility	Repository name: GenBank database, NCBI Data identification number: MZ570560 - MZ570580 Direct URL to data: Accession numbers with hyperlinks were provided in Tables 1 and 2. (https://www.ncbi.nlm.nih.gov/nuccore/?term=MZ570560%3AMZ570580%5Baccn%5D)

**Value of the Data**

• The data provides the diversity of bacterial species, especially *Vibrio, Pseudoalteromonas, Alteromonas*, and *Bacterioplanes* species, found on the surface of marine red algae, *Kappaphycus alvarezii*, cultivated in a closed circulation system.
• The data provides information on the persistent bacteria that adapt to the long-term culture in a closed circulation system.
• The data can serve as a reference for identifying potential symbiotic bacteria associated with *K. alvarezii* in long-term cultures.
• The data is useful for seaweed farmers or practitioners to develop new applications in microbiome-mediated seaweed aquaculture systems.

## 1 Data Description

### 1.1 Blast outputs

This section presents the BLAST outputs of taxonomic identification using the assembled sequences generated by Sanger sequencing. Blast outputs include the bacterial species, accession number of the closest species, accession number of deposited sequences, and percentage of query cover, identities, gaps, and E-value as listed in Tables 1 and 2.

**Table 1:** Blast outputs for the bacterial species found on seaweed culture at day 1 of cultivation.

Bacterial ID	Accession No. of deposited sequences	Bacterial Species	Accession No.of nearest match	Query Cover	Identities	Gaps	E-value
1-D1	MZ570560	*Phaeobacter* sp.	MK801658	99%	99.44%	0%	0.0
2-D1	MZ570561	*Pseudoalteromonas flavipulchra*	KX529477	99%	95.70%	0%	0.0
3-D1	MZ570562	*Bacterioplanes sanyensis*	CP022530	96%	84.99%	2%	0.0
4-D1	MZ570563	*Vibrio alginolyticus*	KY203854	95%	99.34%	0%	0.0
5-D1	MZ570564	*Pseudoalteromonas* sp.	MN889108	98%	99.40%	0%	0.0
6-D1	MZ570565	*Pseudoalteromonas rubra*	NR_026223	96%	97.80%	0%	0.0
7-D1	MZ570566	*Vibrio alginolyticus*	CP054700	98%	99.63%	0%	0.0
8-D1	MZ570567	*Vibrio alginolyticus*	CP054700	98%	99.06%	0%	0.0
9-D1	MZ570568	*Vibrio alginolyticus*	KY203854	99%	99.78%	0%	0.0
10-D1	MZ570569	*Pseudoalteromonas* sp.	MN889151	98%	93.77%	5%	0.0
11-D1	MZ570570	*Grimontia celer*	MW828457	99%	99.06%	0%	0.0

**Table 2:** Blast outputs for the bacterial species found on seaweed culture at day 30 of cultivation.

Bacterial ID	Accession No. of deposited sequences	Bacterial Species	Accession No.of nearest match	Query Cover	Identities	Gaps	E-value
1-D30	MZ570571	*Phaeobacter* sp.	MK801658	94%	98.52%	0%	0.0
2-D30	MZ570572	*Vibrio* sp.	JF682609	100%	98.34%	0%	0.0
3-D30	MZ570573	*Ruegeria* sp.	KJ188015	95%	99.22%	0%	0.0
4-D30	MZ570574	*Bacillus aquimaris*	MK256784	99%	99.19%	0%	0.0
5-D30	MZ570575	*Thalassospira profundimaris*	KJ721943	95%	99.47%	0%	0.0
6-D30	MZ570576	*Alteromonas abrolhosensis*	MT507040	98%	99.41%	0%	0.0
7-D30	MZ570577	*Pseudoalteromonas* sp.	MN889108	99%	99.70%	0%	0.0
8-D30	MZ570578	*Vibrio mediterranei*	MN874182	97%	99.27%	0%	0.0
9-D30	MZ570579	*Bacterioplanes sanyensis*	NR_126264	99%	99.04%	0%	0.0
10-D30	MZ570580	*Alteromonas macleodii*	MT325885	95%	100%	0%	0.0

### 1.2 Colony morphology

**Fig. 1:**
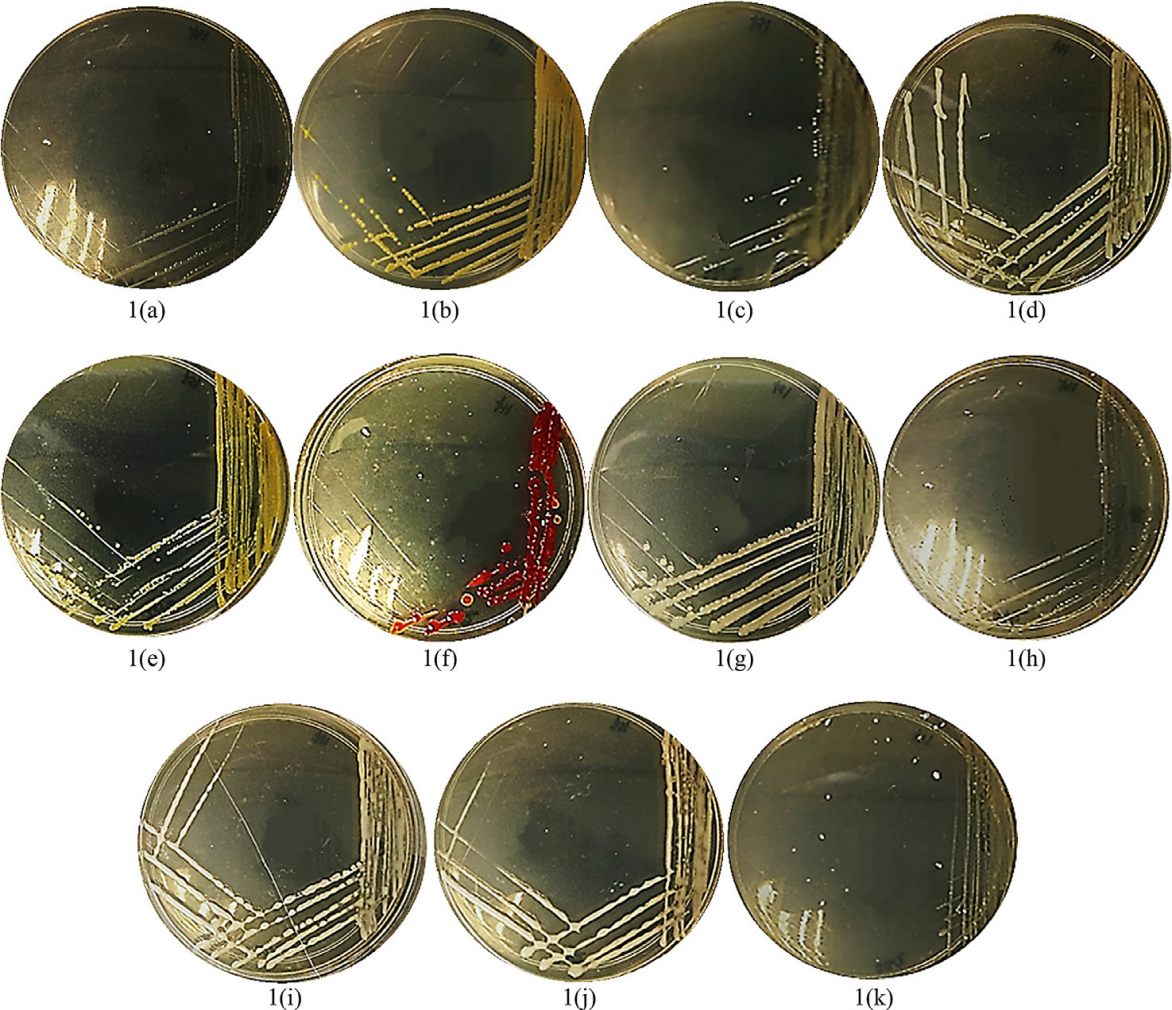
Colonies images of bacterial isolates collected at day 1 of seaweed cultivation. (a) *Phaeobacter* sp.; (b) *Pseudoalteromonas flavipulchra*; (c) *Bacterioplanes sanyensis*; (d) *Vibrio alginolyticus*; (e) *Pseudoalteromonas* sp.; (f) *Pseudoalteromonas rubra*; (g) *Vibrio alginolyticus*; (h) *Vibrio alginolyticus*; (i) *Vibrio alginolyticus*; (j) *Pseudoalteromonas* sp.; (k) *Grimontia celer*.

**Fig. 2:**
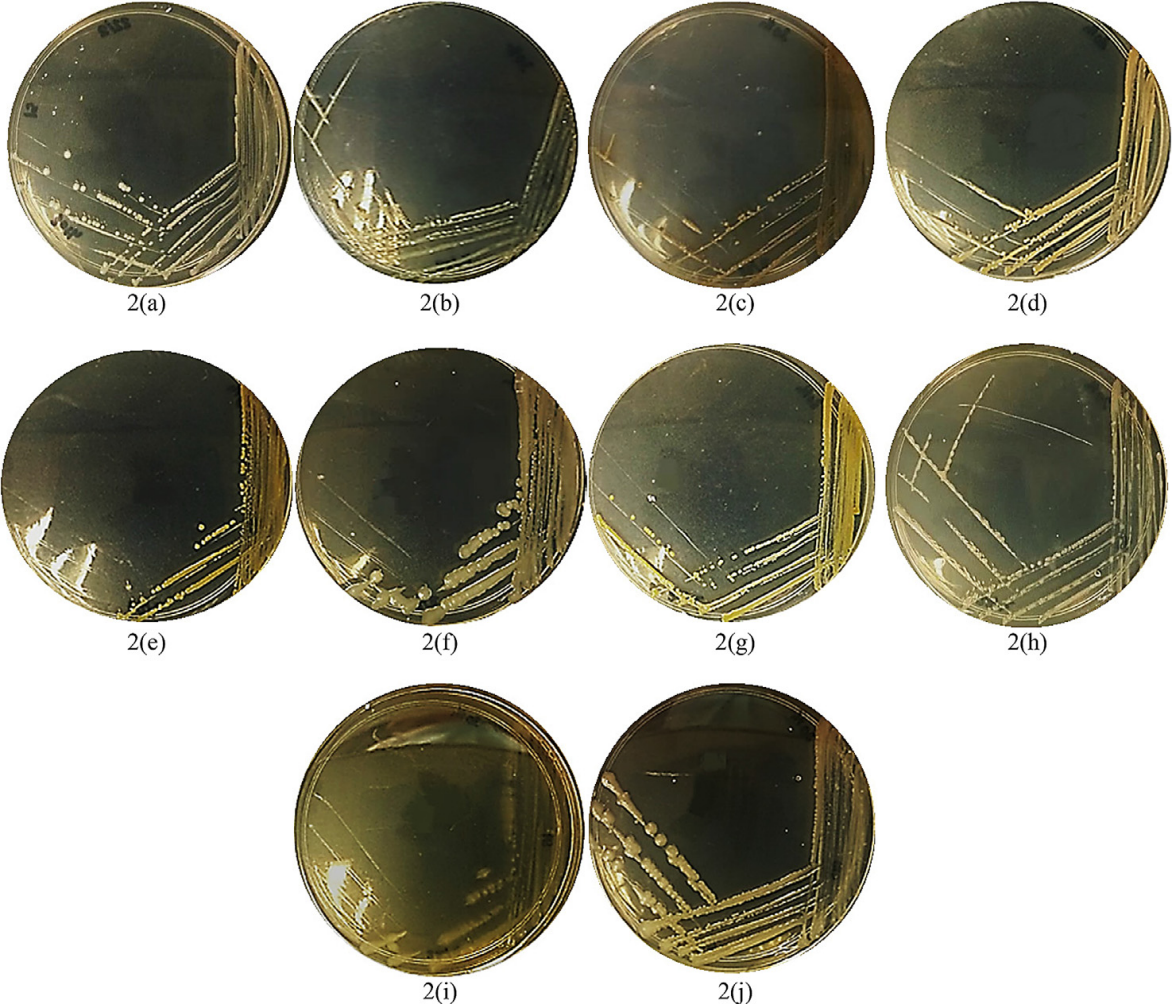
Colonies of bacterial isolates collected at day 30 of seaweed cultivation. (a) *Phaeobacter* sp.; (b) *Vibrio* sp.; (c) *Ruegeria* sp.; (d) *Bacillus aquimaris*; (e) *Thalassospira profundimaris*; (f) *Alteromonas abrolhosensis*; (g) *Pseudoalteromonas* sp.; (h) *Vibrio mediterranei*; (i) *Bacterioplanes sanyensis*; (j) *Alteromonas macleodii.*

Figs. 1 and 2 show the colonies of bacterial species isolated directly from the surface of *K. alvarezii* at the collection and after 30 days of cultivation, respectively. The data revealed isolates within a genus have similar colony morphology except for the *Pseudoalteromonas* genus, where one species, *Pseudoalteromonas rubra*, has a red-pigmented colony compared to other species in the genus. Comparison between the two sets of bacterial groups (Figs. 1 and 2) shows the persistent bacteria that survive throughout the cultivation period. For instance, the genus of *Phaeobacter* sp., *Pseudoalteromonas* sp., *Vibrio* sp., and *Bacterioplanes* sp. were observed to present at both datasets with verification of similar colonies morphology as depicted in the figures.

### 1.3 Construction and analysis of phylogenetic tree

**Fig. 3:**
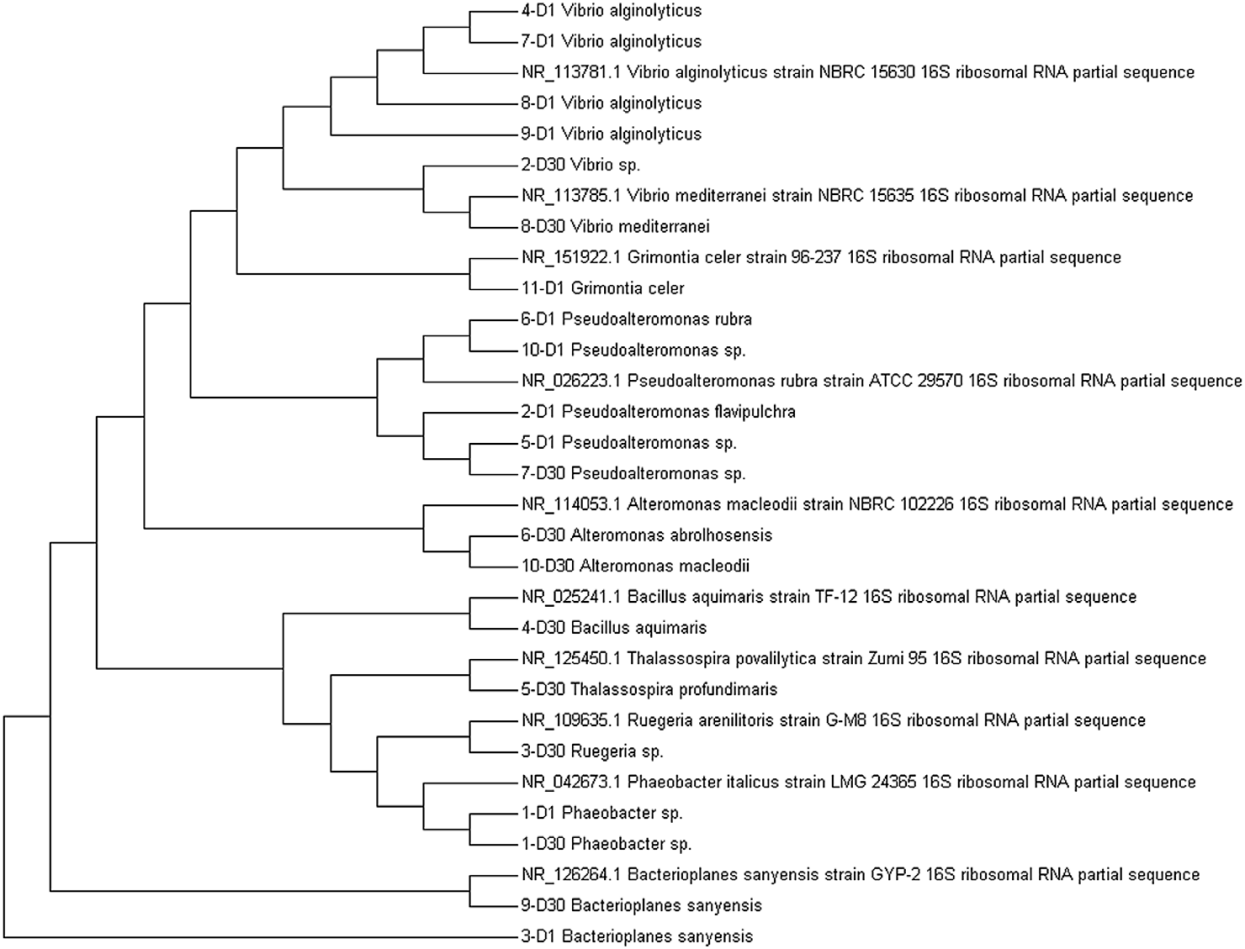
Phylogenetic tree of the bacterial isolates based on the 16S rDNA sequences.

The taxonomy of the 21 isolates was evaluated by constructing a phylogenetic tree based on the 16S rDNA sequences of the isolates and those acquired from GenBank, NCBI. In the phylogenetic tree, the isolates clustered into four major groups: *Vibrio, Pseudoalteromonas, Alteromonas*, and *Bacterioplanes*, as illustrated in Fig. 3. The isolates 4-D1, 7-D1, 8-D1, and 9-D1 were found to be in the same branch as *Vibrio alginolyticus*, with sequence similarities ranging from 98% to 99%. The isolates 2-D30 and 8-D30 belonged to the same branch as *Vibrio mediterranei*, with 98% and 99% similarity, respectively. The isolate 11-D1 was placed in the same group as *Grimontia celer* with 99% similarity. Other isolates, 6-D1, 10-D1, 5-D1, 2-D1, and 7-D30, were clustered in the same branch as *Pseudoalteromonas rubra*, with similarities ranging from 93.15% to 98%. With 84.5% and 99% similarity, the isolates 3-D1 and 9-D30 belonged to the same branch as *Bacterioplanes sanyensis*. The isolates 4-D30, 5-D30, and 3-D30 were found in the same cluster as *Bacillus aquimaris, Thalassospira profundimaris*, and *Ruegeria arenilitoris*, respectively. The sequence similarity of the three isolates was all above 99%. Finally, the isolates 1-D1 and 1-D30 belonged to the same cluster as *Phaeobacter italicus*, with 99.4% and 98.5% similarity, respectively.

## 2 Experimental Design, Materials and Methods

### 2.1 Isolation of marine bacteria

Seedlings were cultivated for 30 days in the seaweed cultivation laboratory at the Biotechnology Research Institute, Universiti Malaysia Sabah. The growth parameters for the seaweed culture were maintained at specific conditions throughout the experiment as described in a previously published method [1]. For marine bacteria isolation, the procedures were performed as reported in a previously published study [2]. On day 1 of seedlings cultivation, seedlings were rinsed thrice with autoclaved seawater to discard any loosely bound epiphyte and other contaminants. Then, sterile cotton buds were used to swab the surface area of three different seedlings under an aseptic condition. The swab samples were then stored in a 50 ml falcon tube containing Phosphate Saline Buffer (PBS) to preserve the bacterial cells in an isotonic environment [3,4]. Similar sampling steps were repeated at day 30 of cultivation. The swab samples for day 1 and day 30 were plated in triplicate on Marine Agar, MA (PENTAIR) plates, and the plates were incubated for five days at 32 °C [5]. During the incubation periods, every new bacterial colony was selected based on its unique morphology, such as the shape, size, color, and texture of the colony, and plated on a new MA plate. These colonies were sub-cultured multiple times to achieve pure cultures with only one type of bacteria. The screening of the pure culture was accomplished by ensuring all the colonies in the new plate were morphologically similar and descended from the same organism. A total of 11 (day 1) and 10 (day 30) isolates were successfully purified into pure cultures and stored in glycerol stocks. All the isolates were kept as stock by adding an aliquot of 1 ml of the overnight culture into a 2 ml cryo-vial containing 1 ml of 50% glycerol for bacterial culture stocks and stored in a −80 °C freezer.

### 2.2 Bacterial DNA extraction

Extraction of bacterial DNA was conducted using the Wizard Genomic DNA Purification Kit (PROMEGA, USA) following the manufacturer's protocols as described in a published procedure [6]. The cells were pelletized by centrifuging 1 ml of overnight culture in a 1.5 ml microcentrifuge tube at 13,000 × g for 2 min. Then, the cells were lysed by adding 600 µl of Nuclei Lysis Solution into the cell mixture and incubating it at 80 °C for 5 min. RNase solution was added into the lysate mixture and incubated at 37 °C for 60 min for RNA removal. The protein precipitation steps were performed by adding 200 µl of Protein Precipitation Solution into the RNase-treated cell lysate and vortexed at high speed for 20 s. Then, the samples were incubated on ice for 5 min and centrifuged at 13,000 × g for another 3 min. For DNA precipitation steps, the supernatant containing DNA was transferred to a clean 1.5 ml microcentrifuge tube containing 600 µl of isopropanol. Then, the tube was gently mixed by inversion until thread-like strands of DNA were visible. After centrifugation, the supernatant was poured off, leaving only the pellet in the tube. Pellet was rinsed at least thrice with 600 µl of 70% ethanol, then centrifuged at 13,000 × g for 2 min and air-dried for 15 min. Lastly, the rehydration process was carried out by immersing the pellet into 100 µl of DNA Rehydration Solution and incubating at 65 °C for 1 h. The tube was gently tapped periodically to mix the DNA mixture. Rehydrated DNA was stored at 4 °C for subsequent downstream applications. The isolated gDNA was monitored by electrophoresis on a 1.5% agarose gel to verify expected bands and purity.

### 2.3 Amplification and sequencing of 16S rDNA

The amplification of the 16S rRNA gene was conducted using DNA Amplification products (Vivantis Technologies, Malaysia) with some modifications [7]. For each reaction, 1.5 µl of DNA template, 1 µl of the forward primer, 1 µl of reverse primer, 5 µl of PCR buffer, 3 µl of MgCl_2_, 1 µl of dNTPs mix, and 0.4 µl of DNA polymerase were added into a tube containing 38.1 µl of double-distilled water. A pair of universal primers, 27 F (5″–AGAGTTTGATCMTGGCTCAG–3″) and 1492 R (5″–GGTTACCTTGTTACGACTT–3″) were used to target nearly full-length of the 16S rRNA gene (∼1400 bp) [8]. The Polymerase Chain Reaction amplification was conducted on a thermal cycler machine (BioRAD PTC 200, USA) under these conditions: 2 min of initial denaturation step at 94 °C, followed by 35 cycles of denaturation (94 °C, 30 s), annealing (60 °C, 30 s), extension (72 °C, 30 s) and final extension (72 °C, 7 min). The PCR products were then held at 4 °C before it was withdrawn and stored at −20 °C freezer. Aliquots of 2 µl of each reaction were resolved on 2% (w/v) agarose gel electrophoresis (AGE) with a 1 kb DNA ladder as a molecular marker in the TBE buffer. Lastly, the generated PCR products of approximately ∼1400 bp were submitted to Apical Scientific Sdn Bhd (First Base Laboratories) for further PCR purification and DNA sequencing in both directions, using the same pair of forward and reverse primers used in the amplification steps.

### 2.4 Sequence analysis

The low-quality regions of 16S rDNA sequences were trimmed, and both sides of the sequenced DNA fragments (forward and reverse) were assembled using DNA Baser Sequence Assembler (version 5.0) downloaded from https://www.dnabaser.com/download/download.html [9]. The trimmed and assembled sequences were converted and arranged into FASTA file format before exporting to the Basic Local website, http://www.ncbi.nlm.nih.gov, for bacterial sequences comparison and taxonomic identification [10]. Trimmed and assembled sequences were deposited in GenBank under accession numbers MZ570560 to MZ570580. All the 21 isolates and their nearest matches were selected for further phylogenetic analysis. ClustalW was used to align all of the sequences, and MEGA7 was used to perform the cluster analysis [11]. The evolutionary distances were computed using the Maximum Composite Likelihood method. The phylogenetic tree was then inferred using the Neighbor-Joining method [12].

## Ethics Statement

The marine red algae *Kappaphycus alvarezii* were provided by Seadling Sdn Bhd (Kota Kinabalu, Sabah, Malaysia). The authors did not use animal or human experimental materials and thus are not subject to ethical concerns.

## CRediT authorship contribution statement

**Rennielyn Rupert:** Conceptualization, Methodology, Software, Formal analysis, Writing – original draft, Writing – review & editing. **Kenneth Francis Rodrigues:** Conceptualization, Methodology, Software, Validation, Writing – review & editing, Supervision. **Harry Lye Hin Chong:** Validation, Resources, Writing – review & editing. **Wilson Thau Lym Yong:** Conceptualization, Methodology, Writing – review & editing, Supervision, Project administration, Funding acquisition.

## Declaration of Competing Interest

The authors declare that they have no known competing financial interests or personal relationships, which have, or could be perceived to have, influenced the work reported in this article.
